# Task-dependent pupillary responses to glossiness and attractiveness judgments

**DOI:** 10.1167/jov.26.9.1

**Published:** 2026-09-01

**Authors:** Hideki Tamura, Shigeki Nakauchi, Tetsuto Minami

**Affiliations:** 1 Department of Computer Science and Engineering, Toyohashi University of Technology, Toyohashi, Japan

**Keywords:** glossiness, attractiveness, pupillary response, temporal principal component analysis, generalized additive modeling

## Abstract

Human pupillary responses are influenced by not only low-level visual properties but also cognitive and affective factors related to task demands. Nevertheless, how the temporal dynamics of pupil modulation differ across evaluative tasks in the context of material perception remains unclear. In this study, we investigated how pupillary responses vary when observers evaluate the same set of object images for either glossiness or attractiveness. These two perceptual attributes were selected to represent distinct cognitive demands: Glossiness is more closely related to lower-level visual processing, whereas attractiveness involves higher-level evaluative processing. The stimuli included 60 grayscale object photographs selected from the THINGS database, representing common real-world items without social or facial content. Low-level image statistics were controlled using histogram matching to equalize luminance and contrast across all the stimuli. Participants viewed each image for 3,000 ms while maintaining central fixation and rated its glossiness or attractiveness on a 7-point scale in separate task blocks. Grand-average waveforms revealed task-dependent modulations of pupil size across rating levels, which is consistent with previous reports that pupillary responses vary with evaluative context even for identical stimuli. Furthermore, temporal principal component analysis and generalized additive modeling revealed that higher glossiness ratings were associated with greater pupil constriction at early, light reflex–like latencies, whereas higher attractiveness ratings elicited greater pupil dilation at later time points. These findings suggest that distinct temporal profiles of pupil size reflect task-specific processing demands in material perception, which is potentially consistent with differences in temporal weighting between visual and affective evaluations.

## Introduction

Humans perceive a rich array of material qualities (*shitsukan*, a Japanese term referring to perceived material attributes such as texture, gloss, and softness) through vision (Adelson, 2001; Anderson, 2011; Anderson & Marlow, 2022; Fleming, 2014; Fleming, 2017; Komatsu & Goda, 2018). These perceptual qualities vary along a continuum, from attributes that are more closely related to physical features (e.g., glossiness [Chadwick & Kentridge, 2015] and surface properties and materials [Fleming, Wiebel, & Gegenfurtner, 2013]) to impressions shaped by affective responses (e.g., preferences, aversions, and emotional valence [Komatsu & Goda, 2018]). Komatsu and Goda (2018) proposed a hierarchical model of material perception, positing that such perceptual attributes are processed along a continuum in the visual system, from relatively lower-level sensory processing to higher-order cognitive evaluation (Komatsu & Goda, 2018). For example, visual features such as glossiness are primarily associated with processing in the ventral visual pathway (Baba, Nishio, & Komatsu, 2021; Komatsu, Nishio, Ichinohe, & Goda, 2021; Nishio, Goda, & Komatsu, 2012; Nishio, Shimokawa, Goda, & Komatsu, 2014; Okazawa, Goda, & Komatsu, 2012), whereas aesthetic impressions such as attractiveness are thought to engage regions such as the prefrontal cortex and reward-related areas (Chatterjee, Thomas, Smith, & Aguirre, 2009; Cinzia & Vittorio, 2009; Kawabata & Zeki, 2004; Kirk, 2008; O'Doherty et al., 2003). In this framework, the perception of glossiness arises from the integration of multiple low-level visual features such as luminance, contrast, and shape (e.g., Anderson & Kim, 2009; Ho, Landy, & Maloney, 2008; Marlow, Kim, & Anderson, 2012; Motoyoshi, Nishida, Sharan, & Adelson, 2007; Nishida, 2019), whereas the perception of attractiveness involves a value-based and emotional interpretation of the same visual input. This hierarchical processing is theoretically supported by psychophysical findings, particularly those involving temporal indicators such as the stimulus exposure and response latency (e.g., Nagai et al., 2015). However, its physiological correlates—especially those captured by psychophysiological indices—remain largely unexplored.

One of the key psychophysiological indicators of internal cognitive and emotional states is pupil size. While pupil diameter is known primarily for its reflexive adjustment to changes in luminance, considerable research has demonstrated that it is also highly sensitive to attentional processes (Binda, Pereverzeva, & Murray, 2013a; Bombeke, Duthoo, Mueller, Hopf, & Boehler, 2016; Mathôt, van der Linden, Grainger, & Vitu, 2013; Naber, Alvarez, & Nakayama, 2013) and emotional arousal (Bradley, Miccoli, Escrig, & Lang, 2008; Kuraguchi & Kanari, 2020; Kuraguchi & Kanari, 2021; Laeng et al., 2013; Oliva & Anikin, 2018). These cognitively driven modulations of the pupil, referred to as psychosensory responses (Ebitz & Moore, 2019; Mathôt, 2018), are believed to involve subcortical structures such as the superior colliculus and locus coeruleus, which regulate early attentional orienting and arousal-related pupil dilation, respectively. In contrast, pupil constriction in response to perceived brightness, even when physical luminance does not change, may reflect the engagement of cortical pathways involved in interpreting surface appearance, although the underlying mechanisms remain unclear. For example, Tamura, Nakauchi, and Minami (2024) reported a significant association between the perceived glossiness of everyday objects and pupil constriction, particularly in the context of the pupillary light reflex (Tamura et al., 2024). This finding suggests that even perceptual qualities associated with visual features, such as glossiness, can modulate the pupillary system depending on the strength of the subjective glossiness impression. Such psychosensory responses involving pupil constriction have also been documented in response to visual illusions such as the glare effect, where perceived brightness is exaggerated beyond physical stimulus properties (Kinzuka, Sato, Minami, & Nakauchi, 2021; Suzuki, Minami, Laeng, & Nakauchi, 2019; Suzuki, Minami, & Nakauchi, 2019; Zavagno, Tommasi, & Laeng, 2017). However, although previous studies have revealed that subjective impressions of visual materials can modulate pupil responses, whether and how different types of perceptual evaluations, such as surface appearance judgments and affective evaluations, which differ in terms of their processing demands, engage distinct pupillary mechanisms remain unclear. While previous work has highlighted the multifactorial nature of pupil responses, including sensitivity to luminance, attention, and affect, relatively little is known about whether these distinct evaluative dimensions are associated with differences in the temporal dynamics of pupil modulation.

To address this research gap, the present study focused on two types of texture evaluation tasks that are assumed to differ in terms of their processing demands: glossiness perception, which is primarily based on physical and visual cues (i.e., a perceptual attribute associated with lower-level visual processing), and attractiveness perception, which involves affective and value-based judgments (i.e., an attribute associated with higher-level evaluative processing). By comparing pupil responses to these distinct evaluative tasks while the same visual stimuli were presented, we aimed to clarify how the level of abstraction or cognitive demand in material perception is reflected in physiological signals. This approach may provide empirical insights into how the perceptual attributes of different abstraction levels are temporally reflected in physiological signals, which is consistent with theoretical reports of material perception, such as those of Komatsu and Goda (2018). In particular, we hypothesize that pupil responses in each task may differ in terms of their temporal characteristics, with glossiness-related responses tending to occur earlier and attractiveness-related responses emerging later. If such temporal dissociation is observed even when identical stimuli are presented and if the magnitude of the change in pupil size depends systematically on the subjective ratings provided in each task, different evaluative dimensions may engage partially distinct cognitive processes, resulting in varied temporal patterns in pupil dynamics. Importantly, our goal is not to directly test the hierarchical model itself but rather to describe and characterize task-specific differences in the pupil response and to explore their neurocognitive implications within this broader theoretical framework.

We tested this hypothesis by conducting a psychophysical experiment in which participants evaluated the same set of visual stimuli under two perceptual tasks, namely, rating glossiness and rating attractiveness, to examine how the temporal profile of pupillary responses varies as a function of task demands. Prior research has demonstrated that even when the same visual input is provided, differences in evaluative goals or cognitive framing can lead to marked changes in pupil dynamics, including task-dependent temporal shifts. For example, Liao, Kashino, and Shimojo (2021) presented participants with the same facial images but instructed them to either provide an attractiveness rating or perform a shape discrimination task (Liao et al., 2021). Their findings revealed transient pupillary constriction that was particularly pronounced during attractiveness judgments. Notably, a similar constriction was observed during shape discrimination. These results suggest that attractiveness-related processing may influence pupil size even in the absence of an explicit evaluation. Similarly, Santos, Fernandes, and Pandeirada (2023) showed participants images of hands holding objects while the evaluative context was manipulated: Participants were told that the hands were either “contaminated and infectious” or “clean” (Santos et al., 2023). Under the contamination framing, participants reported higher levels of disgust and exhibited stronger pupillary constriction. These findings underscore that the task context and evaluative instructions can modulate pupillary responses, highlighting the sensitivity of this physiological measure to cognitive and affective appraisals, even in the absence of changes in visual input.

We conducted a psychophysical experiment in which participants rated identical visual stimuli under two task instructions—glossiness and attractiveness judgment—to explore whether different types of evaluative judgments modulate the temporal characteristics of pupil responses. While both judgments were made on the basis of the same visual input, we expected the associated pupillary responses to exhibit distinct temporal profiles, reflecting differences in processing demands. Specifically, we hypothesized that glossiness judgments would elicit relatively early pupillary constriction consistent with light reflex mechanisms, whereas attractiveness judgments would lead to delayed pupil dilation, which is indicative of emotionally driven arousal responses. We tested this hypothesis using two complementary analytical approaches that capture both localized and global temporal aspects of pupillary dynamics. First, we applied temporal principal component analysis (tPCA) (e.g., Scharf, Widmann, Bonmassar, & Wetzel, 2022) to extract the dominant time-varying components of pupil responses and examined how their peak amplitude and latency varied as a function of the task and subjective ratings. This approach allows for a temporally resolved characterization of how the evaluative context shapes pupil dynamics. For example, Blini, Arrighi, and Anobile (2024) demonstrated that tPCA can identify consistent latent structures—termed “pupil manifolds”—across different tasks and stimuli, suggesting that pupil responses may be shaped by intrinsic physiological constraints (Blini et al., 2024). Building on this insight, we tested whether distinct pupillary components would be obtained when identical stimuli were evaluated under different task instructions. Second, we used generalized additive models (GAMs) to model nonlinear interactions among the task, rating, and time, thereby capturing the full temporal trajectory of pupil size. This method allows us to detect when and how the pupil response diverges across conditions while accounting for individual variability via random effects (Rij, Hendriks, Rijn, Baayen, & Wood, 2019). In particular, the GAMs enabled us to examine not only differences in response magnitude but also the onset and duration of dilation, which are both critical for testing our prediction that attractiveness judgments would be accompanied by delayed, sustained pupil dilation. These two analytical approaches enabled us to examine how distinct types of perceptual evaluation modulate both the timing and structural dynamics of pupillary responses when the same visual input is presented. By directly comparing pupil responses during glossiness and attractiveness judgments of the same object images, we aimed to test whether the evaluative context modulates the temporal dynamics of pupil size. Such task-dependent modulation provides indirect support for the hypothesis that material perception may involve systematic differences in temporal weighting, which is consistent with the hierarchical framework, although we did not directly test this framework.

## Methods

### Participants

Twenty-four naive observers participated in the experiments. The sample size was determined on the basis of a previous study in which the relationship between perceived glossiness and pupillary responses was investigated (Tamura et al., 2024). Participants had normal or corrected-to-normal visual acuity, and the mean age of the participants was 22.4 ± 1.0 years. All experimental procedures were approved by the Institutional Review Board of Toyohashi University of Technology. This study was conducted in accordance with ethical standards and the Declaration of Helsinki. Written informed consent for the publication of participants’ information was obtained from all the participants prior to their participation in the experiment.

### Apparatus

The experiment was conducted in a darkened booth under low ambient illumination conditions (approximately 60 lx). Visual stimuli were presented on a 27-inch LCD monitor (ColorEdge 27 CS2731; EIZO) with a resolution of 1,920 × 1,080 pixels and a refresh rate of 60 Hz, which was calibrated using a SpyderX Elite system (ImageVISION). The monitor was luminance-calibrated such that the luminance of the white point reached 120 cd/m². The participants sat in the booth with their heads stabilized by a chin rest, which ensured a constant viewing distance of 86 cm. Pupil diameter was recorded using an EyeLink Portable Duo eye tracker (SR Research) at a sampling rate of 500 Hz, with a 5-point calibration procedure conducted before each session. The participants provided their responses using a numeric keypad. The stimulus presentation was controlled via MATLAB with Psychtoolbox 3.0 extensions (Brainard, 1997; Kleiner et al., 2007; Pelli, 1997).

### Stimuli

A total of 60 object images were selected from the THINGS database (Hebart et al., 2019; Hebart, Zheng, Pereira, & Baker, 2020), which contains photographs of real-world items commonly encountered in daily life. These images were identical to those employed in our previous study (Tamura et al., 2024). We implemented a multistage selection procedure prior to the main experiment to curate a stimulus set that broadly represents general objects while minimizing potential confounding factors related to glossiness and attractiveness. First, a naive individual who did not participate in the main study categorized 1,854 object names from the THINGS database into three broad semantic categories: objects, creatures, and food. Among these objects, 209 items labeled as creatures and 284 labeled as food were excluded, as we focused on objects that typically have surface materials that are likely to vary in glossiness and attractiveness, which aligned with our experimental goals. Next, the perceived glossiness and luxury of the remaining 1,361 object names were evaluated by the same individual. Luxury was adopted as a more concrete and constrained evaluative dimension during stimulus selection rather than as a direct proxy for attractiveness. This step was intended to encourage grouping on the basis of the semantic and functional characteristics of the objects while reducing the influence of highly idiosyncratic affective or aesthetic impressions. Although luxury and attractiveness are conceptually distinct, luxury was used as a constrained evaluative dimension during stimulus selection. On the basis of this preliminary categorization, 60 object images were selected such that the glossiness and attractiveness distributions were approximately uniform across the set. This strategy helped prevent unintentional clustering in either dimension and allowed the two rating axes to be manipulated independently during the main task. An additional analysis of the final stimulus set confirmed that images categorized as high in luxury received higher attractiveness ratings than images categorized as low in luxury did (*r* = 0.53, *p* < 0.001), indicating that the two evaluative dimensions were substantially related while remaining conceptually distinct. Each selected image depicted a single, clearly visible object with a sufficiently large surface area available for visual evaluation. Each of the 60 selected images was drawn from a distinct category defined in the THINGS database. The images were resized to 512 × 512 pixels, converted to grayscale, and cropped to remove the background while preserving the main object. We applied histogram matching using the SHINE toolbox (Willenbockel et al., 2010) to control for low-level visual features, and the luminance histograms of all images were aligned to the average across the set. Through this procedure, we equalized key image statistics, resulting in the following matched values: mean luminance = 27.24 ± 0.68 cd/m², standard deviation of the pixel luminance values = 28.20 ± 0.69 cd/m², skewness = 1.25 ± 0.06, and kurtosis = 3.71 ± 0.20.

### Procedure

**Figure 1.**
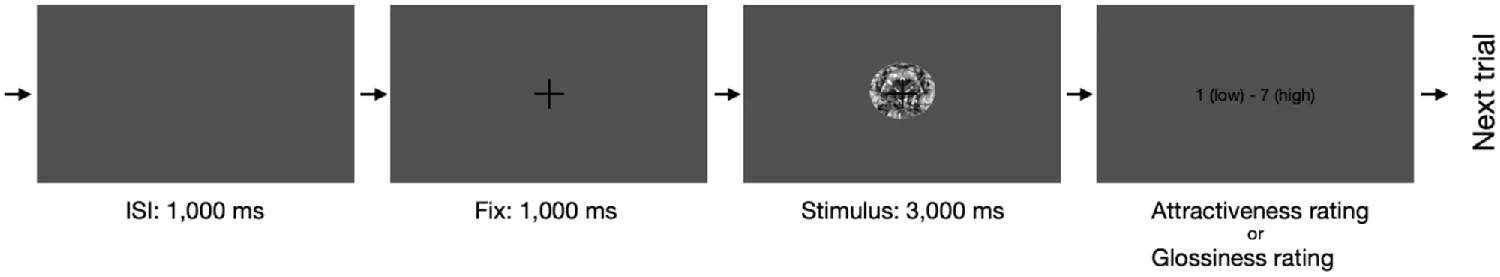
Procedure. Schematic showing the sequence of a single trial in the experiment. Note that the relative sizes of the fixation point and stimulus with respect to the screen are not to scale and differ from those used in the actual experiment.

The experimental procedure is illustrated in Figure 1. Each trial began with a 1,000-ms interstimulus interval (ISI), followed by the presentation of a black fixation cross (0.6 × 0.6 degrees, 0.15 cd/m²) on a gray background (9.19 cd/m²) for 1,000 ms. A stimulus image then appeared at the center of the screen, together with the fixation cross, and remained visible for 3,000 ms. Participants were not allowed to respond during stimulus presentation, and responses were collected only after the stimulus was removed. Each stimulus depicted a single object positioned within a 6 × 6–degree region of the visual field. The participants were instructed to rate the object using a 7-point Likert scale (1 = low score, 7 = high score). Two types of rating tasks were employed: one in which perceived attractiveness was assessed and another in which perceived glossiness was assessed. In both tasks, the participants were instructed to base their ratings on their personal impressions rather than on any explicit objective criteria, although glossiness judgments are generally more closely related to image-based cues than attractiveness judgments are. The participants entered their responses using a numeric keypad. The next trial began immediately after a response was provided. The participants were asked to maintain central fixation whenever the fixation cross was visible and to blink only during the ISI period when the screen was blank. Each participant completed a total of 240 trials, comprising 60 images × 2 rating tasks × 2 repetitions. The two rating tasks were administered in separate blocks of 120 trials each. The order of these blocks was counterbalanced across participants, and the trial order within each block was randomized. A 5-point calibration of the eye-tracking device was performed at the beginning of each block. The experiment was conducted in a booth with constant ambient illumination throughout the session, and no explicit dark-adaptation period was introduced before each block. Additionally, the participants were allowed to take self-paced breaks between blocks.

### Data analysis

Behavioral and pupillometric data were analyzed using R (version 4.3.3) and MATLAB R2023a. We computed the mean attractiveness and glossiness ratings for each image on the basis of the participants’ responses. Only trials retained for the pupillometric analysis were included; trials that were excluded because of signal artifacts or missing data were also omitted from the behavioral analysis to ensure consistency.

Pupil diameter was analyzed from the onset of the fixation cross to the end of the stimulus presentation. Preprocessing was performed using procedures established by Nakakoga et al. (2021) and Tamura et al. (2024), with data from the right eye used in the analysis. Eye blinks were identified as time intervals in which the pupil velocity exceeded 0.011 mm/ms. These intervals, along with 10-ms buffers on either side (i.e., a total of 20 ms), were excluded from the analysis. The missing segments were interpolated using a shape-preserving piecewise cubic spline function. Entire trials were excluded from the analysis if they contained a blink lasting longer than 1 s, if more than 30% of the data points were missing, or if the pupil signal was absent at either the beginning or end of the trial. Participants for whom more than 50% of the trials were rejected were selected to be excluded on the basis of preestablished criteria; however, no participants met this threshold. Consequently, the data from all 24 participants were retained for analysis (mean rejection rate: 12.07% ± 13.98%). Baseline correction was performed by subtracting the average pupil diameter measured during the 200 ms preceding stimulus onset. The baseline-corrected data were smoothed using a 10-ms moving average filter to reduce high-frequency noise.

Pupillary responses were first averaged across trials for each task and rating level to obtain grand-average waveforms. In addition, tPCA was conducted using the method proposed by Scharf et al. (2022). The analysis was performed on the covariance matrix of the pupil time series, and unrotated factor loadings were first estimated. The final factor solution was then obtained using Geomin rotation. The analysis was conducted over the full stimulus presentation period (0–3,000 ms relative to stimulus onset). The resulting factor loadings were multiplied by the original pupil data to compute the pupil component scores using an approach analogous to that described by Wetzel, Einhäuser, and Widmann (2020).

GAMs were then applied to the component scores for each factor to further examine the continuous effects of task on pupillary dynamics. The response variable was the component-specific pupil time series obtained by weighting the original pupil data with the loading of each temporal principal component rather than the raw pupil diameter itself. Temporal effects were modeled using smooth functions of time that were estimated separately for each combination of task and rating, allowing the temporal trajectory of the pupil response to vary flexibly across conditions. Participant-level variability was considered by including random intercepts for each participant. GAMs were fitted using smoothing parameters that were estimated using a restricted maximum likelihood approach. A discrete approximation strategy was employed to increase computational efficiency given the large number of observations. The basis dimension for the time-smoothing parameters was set to k = 20. The adequacy of this choice was confirmed using the gam.check function, which indicated the sufficient flexibility of the smoothing terms. Confidence intervals were derived from the model-based standard errors of the estimated smoothing parameters.

## Results

### Behavioral results

**Figure 2.**
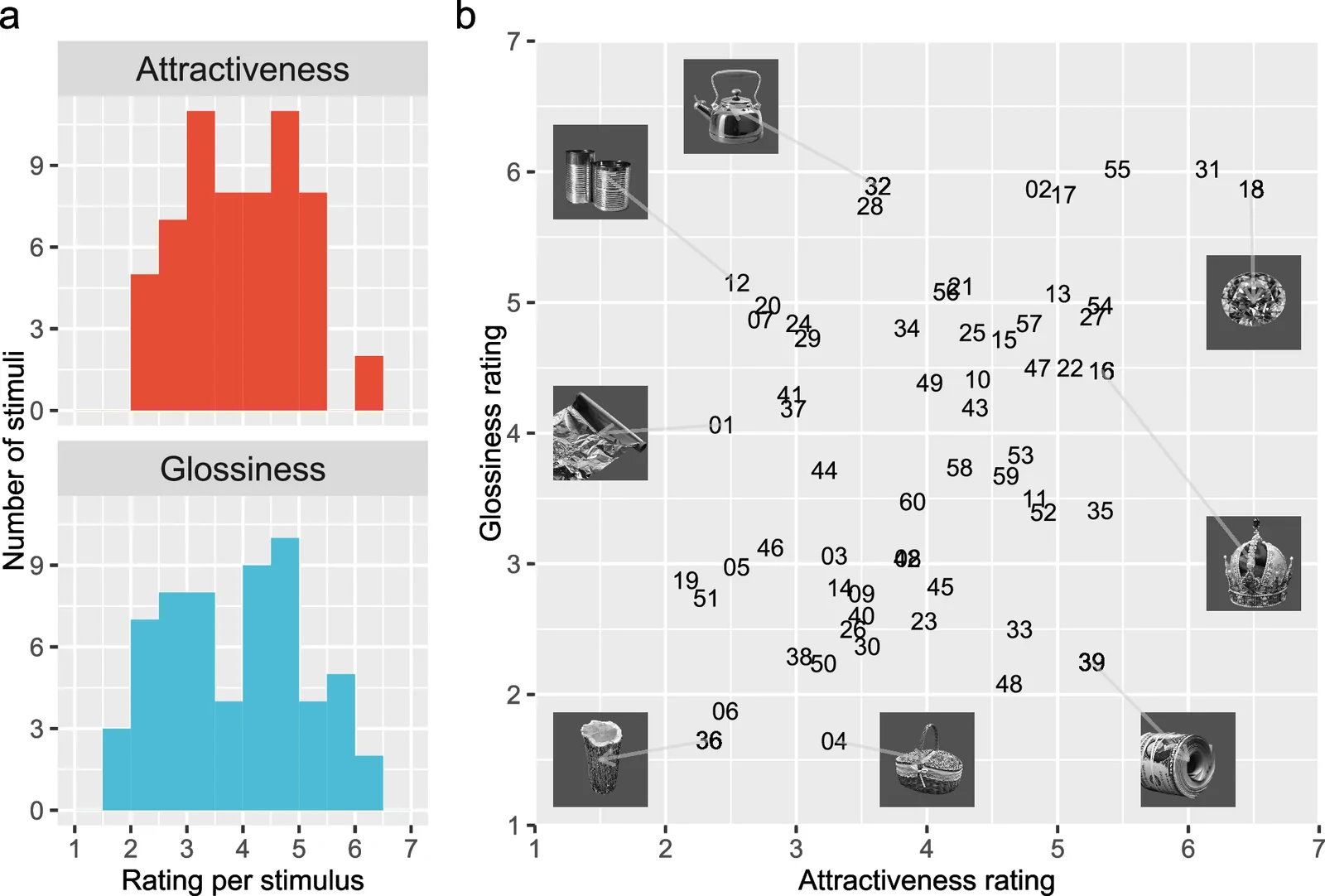
Ratings in the experiment. (**a**) Histogram showing the mean ratings for the 60 stimuli. The horizontal axis indicates the continuous image-wise mean rating values (range: 1–7), and the vertical axis indicates the frequency. Histograms were constructed using fixed bin edges with a bin width of 0.5. The colors represent the rating tasks (red: attractiveness; blue: glossiness). (**b**) Scatterplot of the mean ratings for the 60 stimuli. The horizontal axis indicates attractiveness, and the vertical axis indicates glossiness. The numbers correspond to the stimulus IDs.

Histograms of the mean attractiveness and glossiness ratings for each image are shown in Figure 2a. The attractiveness ratings had a relatively broad distribution, with two local peaks, whereas the glossiness ratings exhibited a bimodal distribution with peaks at approximately 3 and 5. The average attractiveness and glossiness ratings across the 60 images were 3.97 ± 1.03 and 3.90 ± 1.24, respectively. The two ratings were positively correlated (*r* = 0.42, 95% CI [0.19, 0.61], *t*(58) = 3.53, *p* < 0.001), indicating that stimuli rated as more attractive also tended to be rated as more glossy. The distribution of the ratings for each stimulus in the two-dimensional space of attractiveness and glossiness is shown in Figure 2b. Objects rated high in both dimensions included gemstones (stimulus ID = 18) and precious metals (16), whereas those rated low in both dimensions included wood (36) and wicker baskets (04). In contrast, stimuli such as bundles of cash (39) were rated as high in attractiveness but low in glossiness, whereas items such as metal cans (12) were rated as low in attractiveness but high in glossiness.

### Pupil responses: Grand average

**Figure 3.**
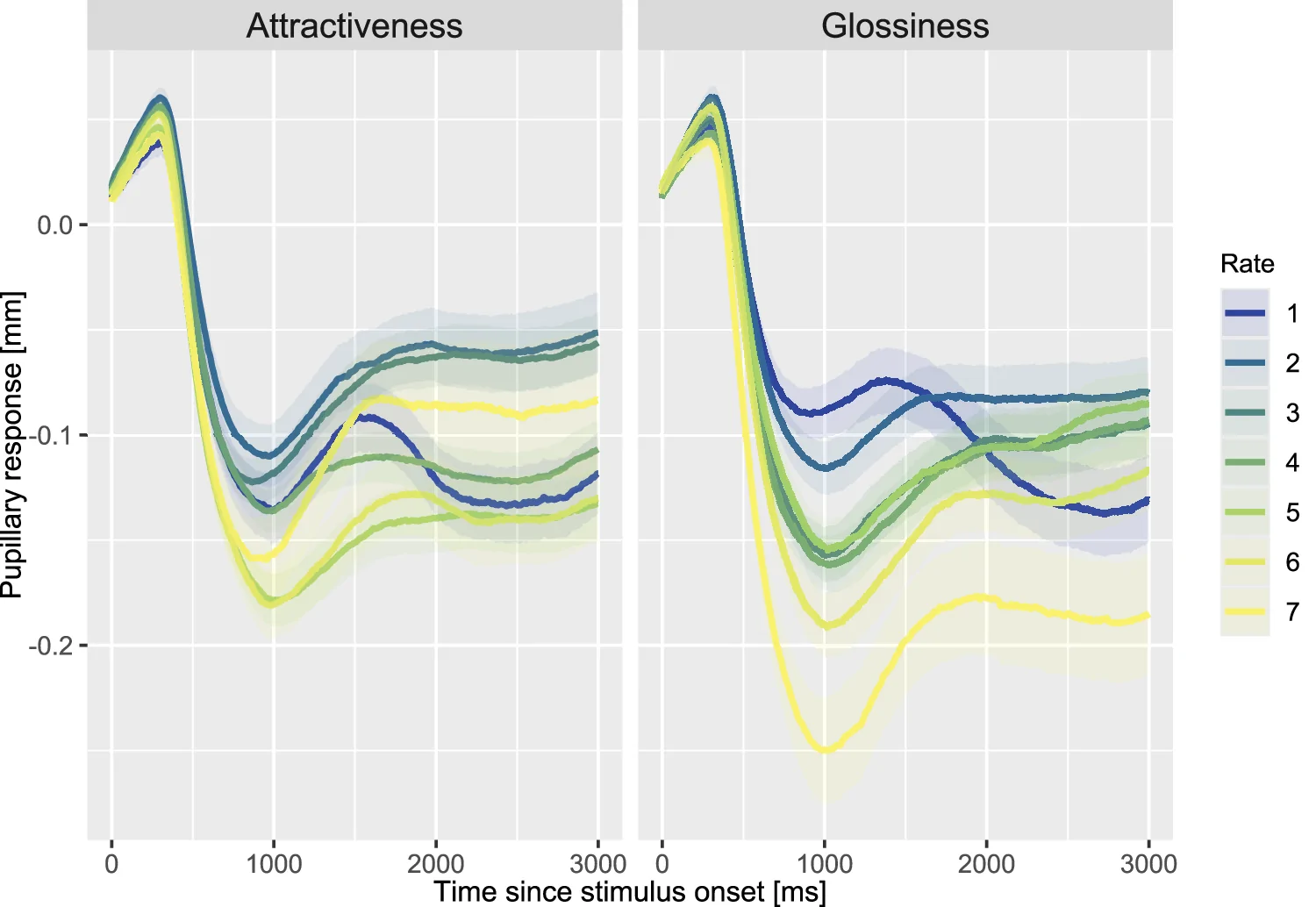
Average pupillary responses. The horizontal axis indicates the time from stimulus onset, and the vertical axis indicates the mean pupil response. Each color corresponds to a different rating level, and the shaded error bars represent the standard error of the mean.

The average pupil responses for each rating level across the two tasks are shown in Figure 3. Although the same set of stimuli was used in both tasks, the pupil responses varied depending on the task. The peak of pupil constriction occurred approximately 1,000 ms after stimulus onset. Furthermore, differences in mean pupil responses across rating levels were observed in both tasks. Specifically, in the attractiveness task, intermediate ratings were associated with greater pupil constriction, whereas in the glossiness task, higher ratings were associated with stronger pupil constriction.

### Pupil responses: Temporal PCA

**Figure 4.**
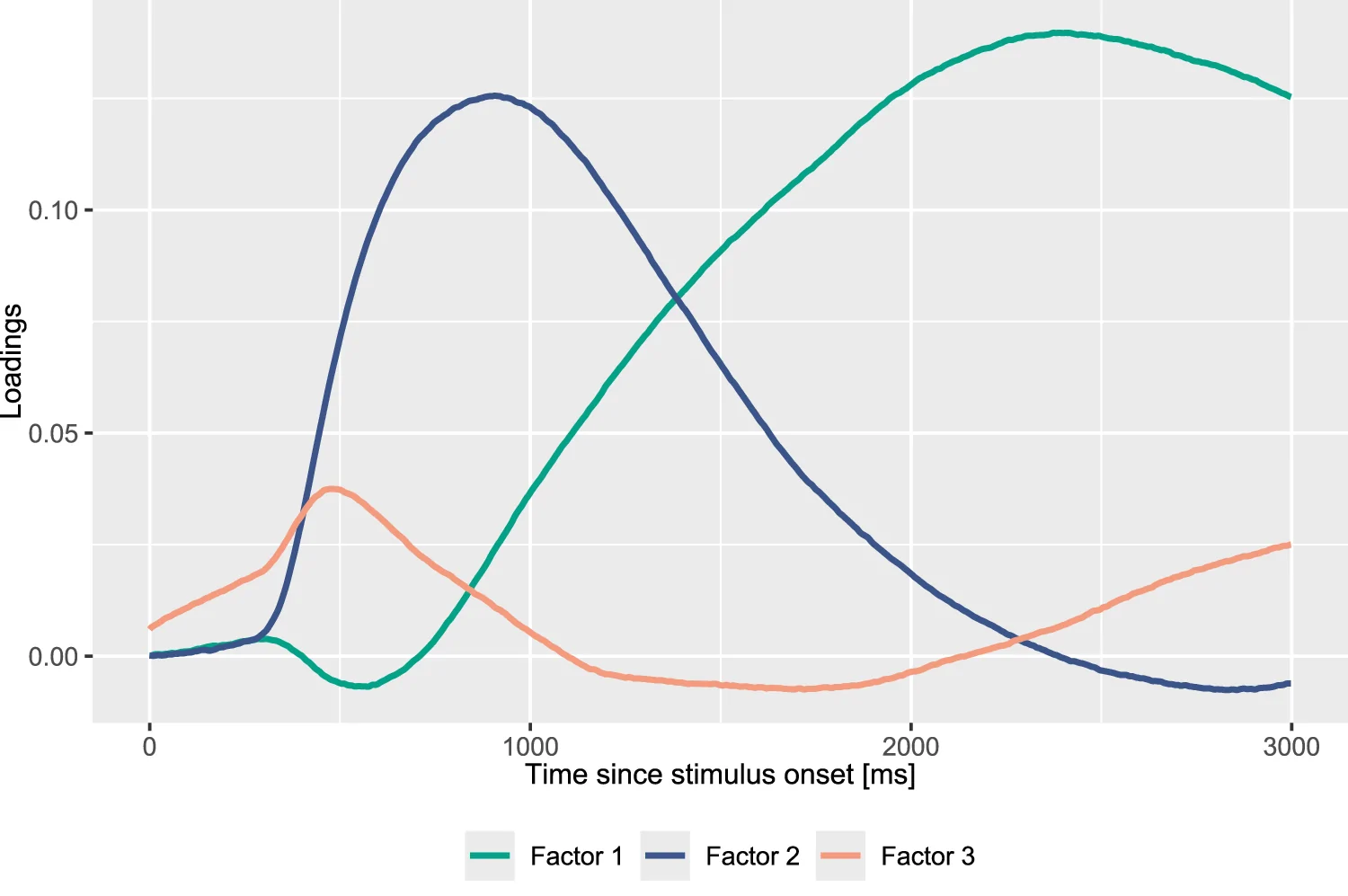
tPCA factor loadings. The horizontal axis indicates the time since stimulus onset, and the vertical axis indicates the factor loading. The three colored lines correspond to Factor 1 (green), Factor 2 (blue), and Factor 3 (orange).

The temporal components that primarily accounted for the observed dynamics were not fully captured by the grand-average pupil responses across trials and rating levels. Therefore, we applied tPCA to the raw pupil time-series data using established procedures to characterize the temporal dynamics of the pupil responses (Scharf et al., 2022). The resulting factor loadings are shown in Figure 4. Since the first three components collectively accounted for 97.7% of the total variance, we limited our subsequent analyses and interpretations to these components. Factor 1 (72.5%) captured a late component characterized by a temporally broad and sustained response. Factor 2 (15.6%) reflected an earlier component with a more temporally confined profile that peaked approximately 1,000 ms after stimulus onset, consistent with the canonical pupillary light reflex. Factor 3 (9.6%) represented a component that peaked even earlier and exhibited a transient profile, likely reflecting an orienting response associated with early sensory-driven processes.

**Figure 5.**
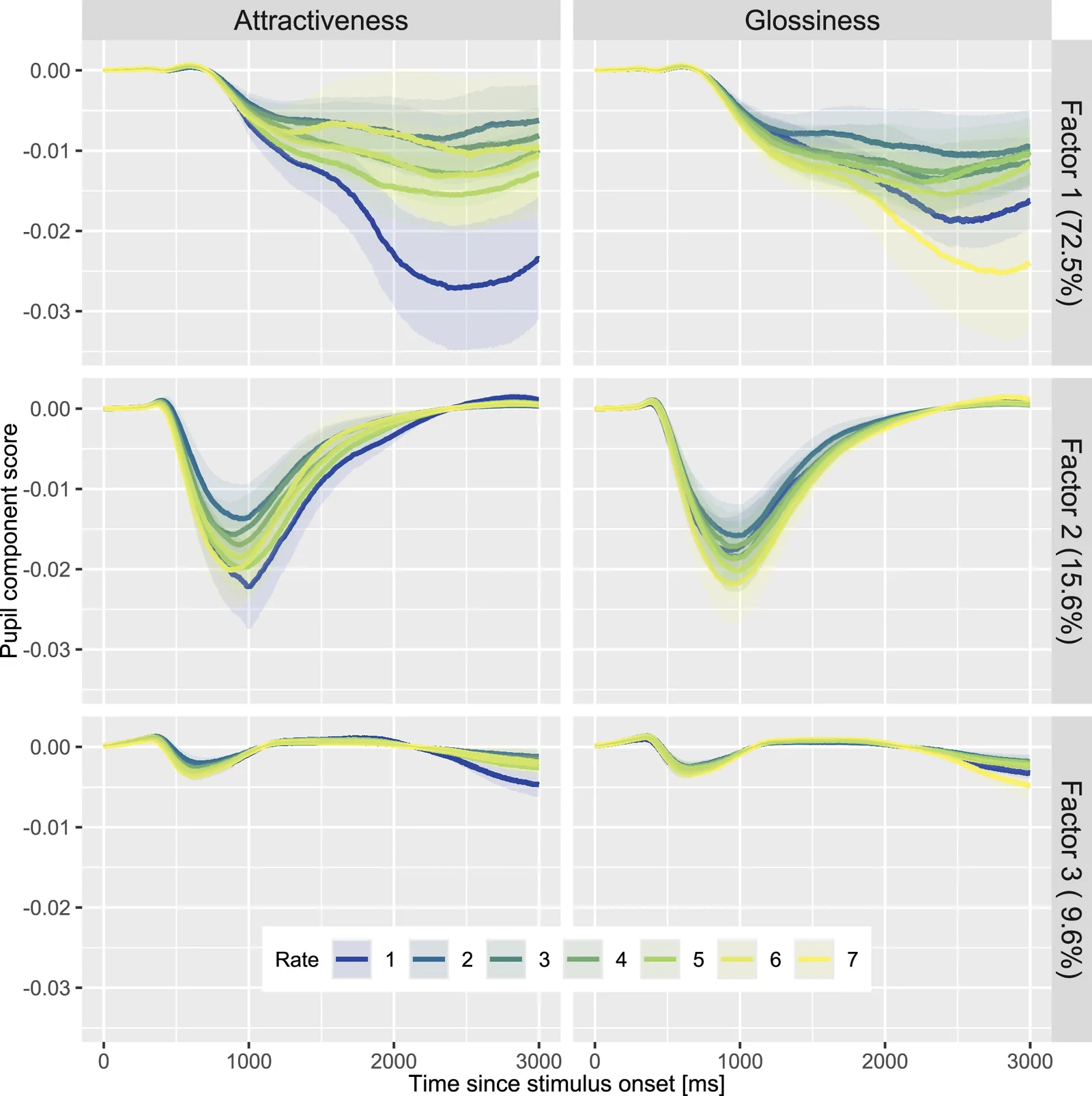
Pupil component scores derived from tPCA. The horizontal axis indicates the time since stimulus onset, and the vertical axis indicates the pupil component score. Each color corresponds to a different rating level, and the shaded error bars represent the standard error of the mean. The rows represent the three factors derived from tPCA, and the columns represent the two rating tasks.

Next, using an approach described in a previous study (Wetzel et al., 2020), we computed single-trial component scores by projecting each single-trial pupil time series onto the factor loadings derived from tPCA. The results are presented in Figure 5, with the colors indicating the rating task and subjective rating value.

As shown in Figure 5, Factor 1 appeared to be systematically modulated by the attractiveness ratings. Specifically, a more sustained pupil constriction following the light reflex was observed in trials in which the participant provided a low attractiveness rating, whereas greater pupil dilation during the later time window was observed in trials in which the participant provided a high attractiveness rating.

In contrast, systematic rating-dependent patterns were observed for both Factor 1 and Factor 2 during the glossiness task. With respect to Factor 1, higher glossiness ratings were associated with stronger pupil constriction after the light reflex. With respect to Factor 2, which corresponds to the timing of the light reflex, increased pupil constriction was associated with higher glossiness ratings. Notably, the magnitude of pupil constriction varied systematically with the level of perceived glossiness, indicating a parametric relationship between these factors.

We further quantified the relationship between factor scores and rating levels by conducting linear mixed-effects model (LMM) analyses on the trial-averaged factor scores (averaged within each condition). Across Factors 1–3, no significant main effects of the task or task × rating interaction were observed. A significant main effect of the rating was observed only for Factor 2 ($F\left(1.00,\;3,241.94\right)=10.61,p=0.001,\eta_{p}^{2}=0.003$), whereas no such effect was observed for Factor 1 or 3.

Additionally, we analyzed the reaction time (RT) as a function of the task and rating to assess whether the observed factor–rating patterns could be explained by decision difficulty or uncertainty (Supplementary Figure S2). The RT showed a pronounced inverted-U relationship with the rating level, with slower responses for intermediate ratings, particularly in the attractiveness task. However, this pattern did not mirror the task-dependent modulation observed in the pupillary responses, suggesting that the pupil responses cannot be explained solely by differences in decision difficulty.

### Generalized additive model

**Figure 6.**
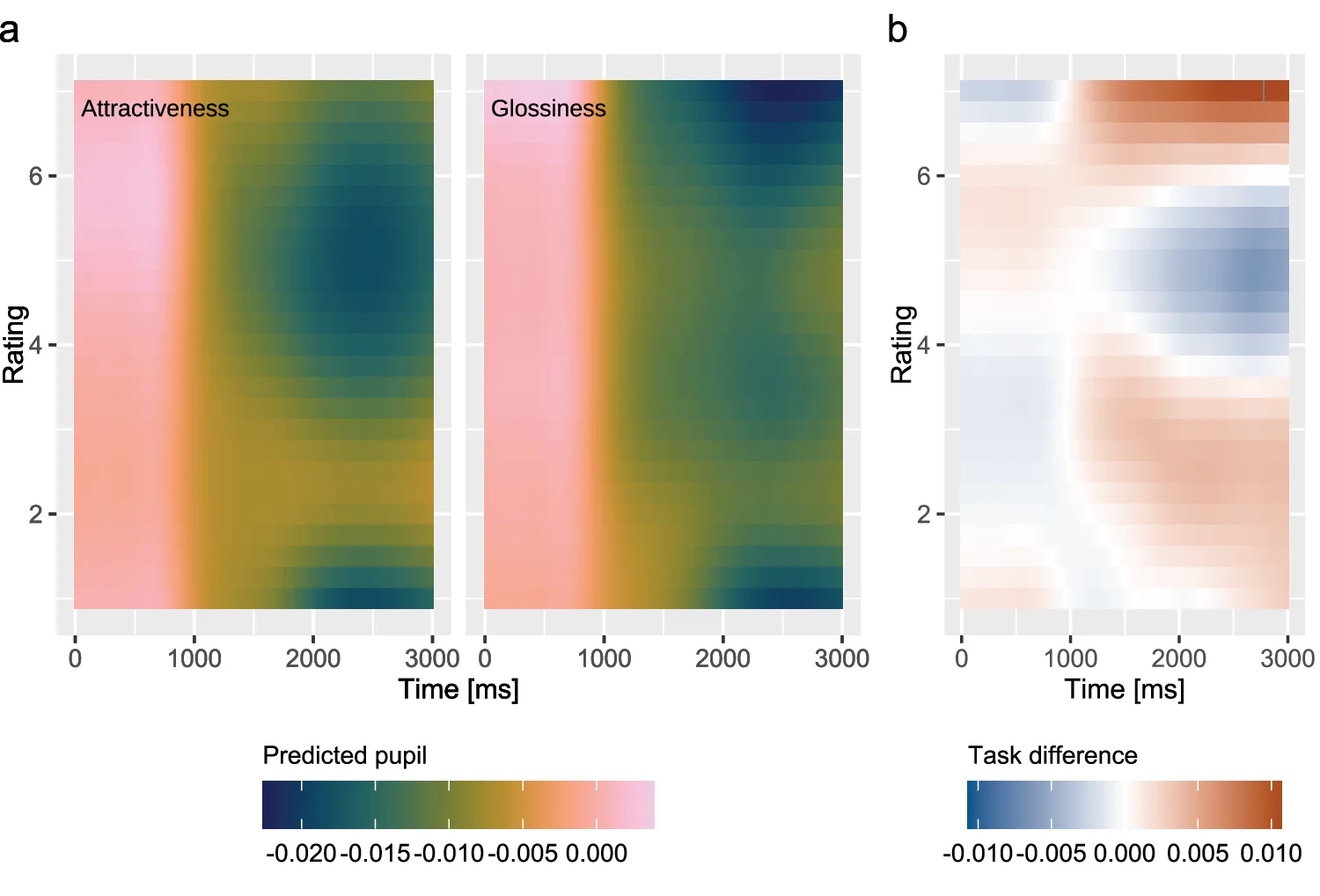
Predictions of the generalized additive model for Factor 1 across the continuous time × rating space. (**a**) Predicted pupil component scores are shown separately for the attractiveness and glossiness tasks. (**b**) Difference map showing attractiveness scores minus glossiness scores across the same time × rating space. Warm colors indicate relatively larger predicted values in the attractiveness task, whereas cool colors indicate relatively larger predicted values in the glossiness task.

We next examined GAM-predicted pupil responses across the continuous time × rating space to characterize how pupil responses varied as a function of both time and rating level. The pupil responses, which were predicted separately for the attractiveness and glossiness tasks, for Factor 1 are shown in Figure 6a (the responses for Factors 2 and 3 are shown in Supplementary Figures S3 and S5, respectively). In both tasks, the pupil responses exhibited a characteristic temporal profile, with initial pupil constriction followed by gradual recovery. The magnitude and temporal evolution of this response varied systematically with the rating level.

The task difference map (attractiveness–glossiness) across the same time × rating space is shown in Figure 6b to directly compare the responses in the two tasks. Task-dependent modulation was observed after approximately 1,000 ms, with distinct regions showing positive and negative differences depending on the rating level. Specifically, higher ratings tended to be associated with stronger responses in the attractiveness task, whereas intermediate ratings tended to be associated with relatively stronger responses in the glossiness task. These results indicate that pupil responses are not only modulated by the rating level during each task but also differ systematically between tasks in a time- and rating-dependent manner.

**Figure 7.**
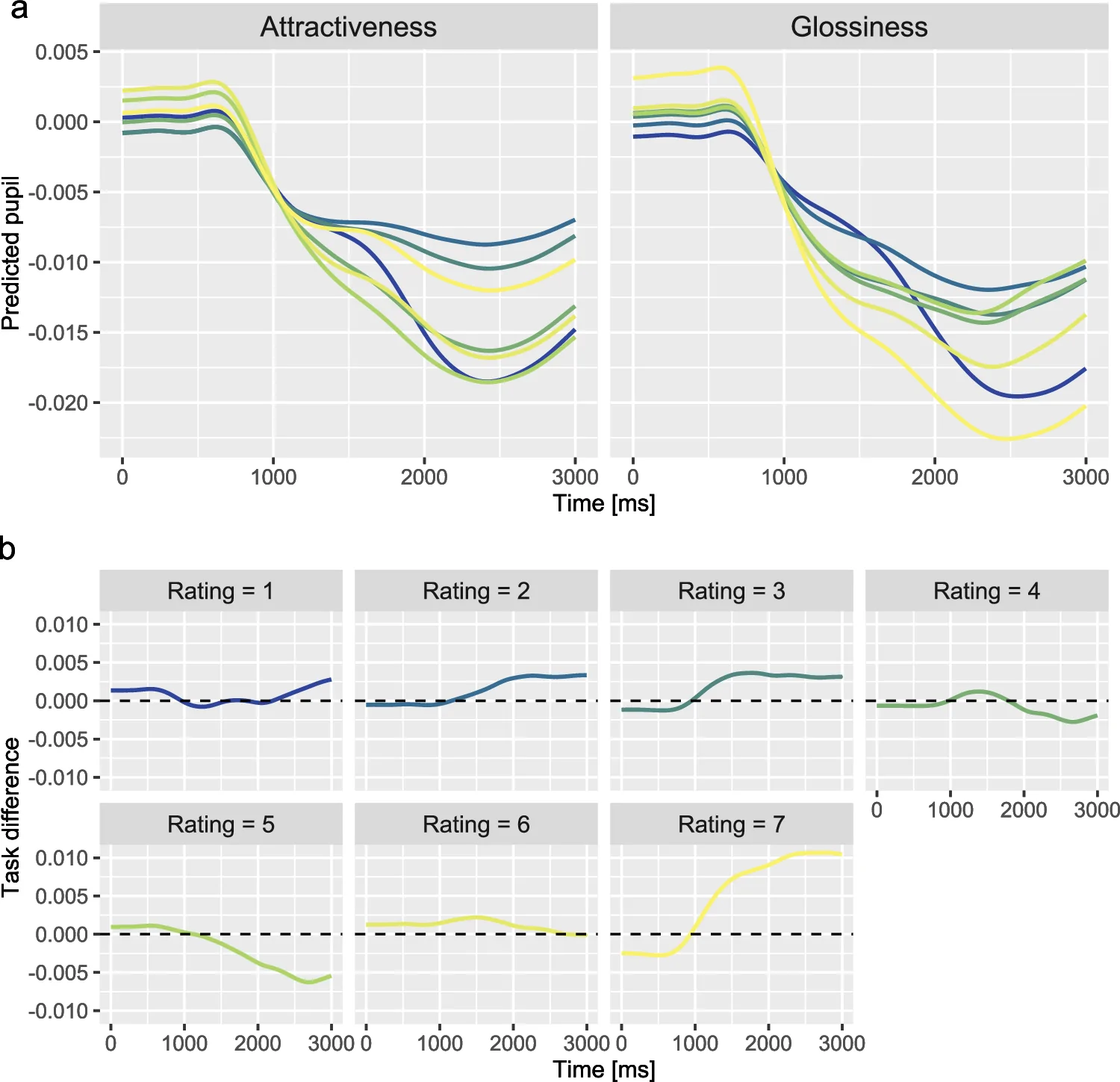
Time-resolved modulation of pupil responses as a function of the rating and task. (**a**) Line-slice representations of the predicted pupil responses (Factor 1) over time for each rating level (1–7); the results are shown separately for the attractiveness and glossiness tasks. Within each task, the temporal profile of the pupil response varies systematically with the rating, indicating the continuous modulation of pupil dynamics across the rating range. (**b**) Task differences (attractiveness–glossiness) in each rating level, shown as time-resolved line plots. Positive values indicate stronger responses in the attractiveness task, whereas negative values indicate stronger responses in the glossiness task. The direction and magnitude of the task differences vary across both time and rating level, showing a structured task × rating × time interaction rather than effects confined to a specific rating level.

Additionally, we visualized line-slice representations of the predicted pupil responses for each rating level to examine how temporal dynamics change across rating levels (Figure 7 for Factor 1; the results for Factors 2 and 3 are shown in Supplementary Figures S4 and S6, respectively). Within each task, the temporal profile of the pupil response varied continuously as a function of the rating (Figure 7a). In the attractiveness task, higher ratings were associated with later dilation, whereas lower ratings were associated with relatively attenuated responses. In contrast, a different pattern was observed in the glossiness task, with intermediate ratings associated with relatively stronger modulation. Furthermore, we visualized the task difference (attractiveness–glossiness) for each rating level to directly compare the responses in the two tasks (Figure 7). These difference profiles revealed that both the magnitude and direction of the task effects varied systematically across time and ratings. In particular, higher ratings were associated with positive differences, whereas intermediate ratings were associated with negative differences, indicating a systematic interaction between the task and rating. These results indicate that the observed task differences cannot be attributed to a specific rating level and instead reflect a continuous and structured modulation of temporal dynamics across the entire rating range.

**Figure 8.**
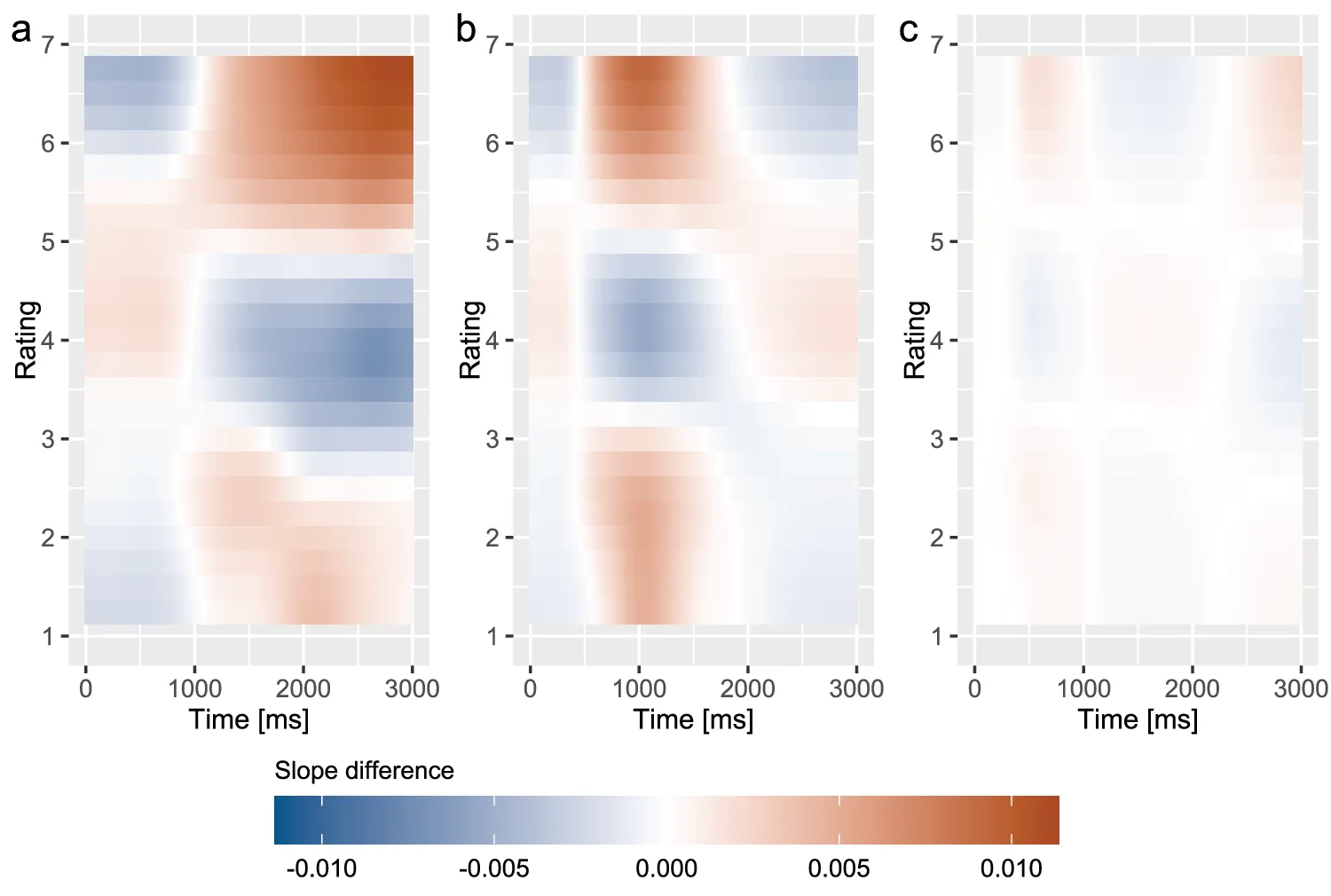
Time-resolved task-dependent modulation of rating sensitivity across temporal components. Slope difference maps (attractiveness–glossiness) are shown across the continuous time × rating space for each tPCA-derived component: (**a**) Factor 1, (**b**) Factor 2, and (**c**) Factor 3. Differences in slope were computed as the derivative of the predicted pupil responses with respect to the rating, reflecting the strength of the rating-dependent modulation within each task. Positive values indicate stronger rating sensitivity to the ratings in the attractiveness task, whereas negative values indicate stronger sensitivity to the ratings in the glossiness task.

Furthermore, we computed time-resolved differences in slope (attractiveness–glossiness) across the time × rating space (Figure 8) for each factor to quantify task-dependent differences in rating sensitivity over time. Factor 1 was associated with pronounced slope differences that extended across the time × rating space, particularly at later time points, indicating substantial task-dependent modulation of rating sensitivity. Factor 2 was associated with more localized and temporally earlier differences in slope, suggesting more limited and possibly stimulus-driven modulation. In contrast, Factor 3 was associated with minimal differences in the slope across the entire time × rating space.

These descriptive patterns were supported by the results of the GAM analysis, which revealed significant effects of the rating and its interaction with time in both tasks (all *p* values <0.001), indicating that the pupil responses were systematically modulated by the rating level in a time-dependent manner. Taken together, these results indicate that task-dependent modulation of pupil responses is primarily expressed in later temporal components, with weaker or negligible effects observed for earlier and less structured components. Overall, these analyses provide a comprehensive characterization of the task × rating × time interaction, demonstrating that pupil responses are continuously modulated by both the rating level and task demands.

## Discussion

### Overview of the main findings

In this study, we compared pupillary responses between two evaluative tasks (glossiness and attractiveness ratings) performed with identical visual stimuli. The rating data confirmed that participants perceived a broad range of glossiness and attractiveness across the images, ranging from low to high values, indicating that the experimental manipulation was successful. Grand-averaged pupil traces revealed a common pattern of light-induced constriction followed by a gradual return to baseline; however, the amplitude and temporal characteristics of this response varied as a function of the task and subjective rating. tPCA revealed distinct pupil response components that differed systematically across factors, tasks, and rating levels. Furthermore, GAMs of these components revealed consistent task-dependent differences in pupil dynamics. Taken together, these findings indicate that pupillary responses are modulated by not only the physical properties of the stimulus but also the evaluative context of the task and the subjective rating assigned to the same visual input.

### Behavioral characteristics and relation to previous studies

Few behavioral studies have concurrently assessed physically grounded and affect-laden texture qualities, such as glossiness and attractiveness, using the same set of visual stimuli, as illustrated in Figure 2a. One notable exception is the study by Fleming et al. (2013), in which participants were asked to rate texture images of everyday objects using various descriptive adjectives (Fleming et al., 2013). They reported that interparticipant correlations were relatively high (approximately 0.6–0.8) for physically grounded material attributes (e.g., glossiness and transparency) but substantially lower (approximately 0.3) for more affect-laden impressions such as prettiness. Consistent with this trend, in the present study, interparticipant correlations for glossiness and attractiveness were 0.53 and 0.41, respectively. These results suggest that physically grounded texture impressions tend to be more consistently perceived across observers and are more closely related to the optical properties of the object itself, whereas affect-laden impressions such as attractiveness are more susceptible to individual differences and subjective interpretation.

Using the overall average pupil data, we confirmed the main finding of Tamura et al. (2024), who reported a systematic relationship between subjective glossiness ratings and pupil responses to everyday objects (Figure 3). Consistent with their results, higher glossiness ratings were associated with greater pupil constriction, confirming the previously reported relationship.

### Task-dependent temporal weighting of pupil responses

While Tamura et al. (2024) primarily summarized pupillary responses using a single index, namely, the magnitude of the maximum pupil constriction, we extended this approach by applying time-resolved methods such as tPCA and GAMs. This analytical advancement allowed us to examine the full temporal dynamics of the pupil response without relying solely on peak amplitude or latency measures. The tPCA and GAM results revealed that even when the same set of visual stimuli was used across conditions, the temporal profile of the pupillary response systematically varied as a function of the evaluative task. Importantly, this time-resolved modeling approach allowed us to characterize the continuous interaction among the task, rating, and time rather than relying on discrete comparisons at specific rating levels or time points.

As illustrated in Figure 5, each factor appears to capture temporally distinct components of the pupillary response: Factor 1 (F1) corresponds to a late dilation-related component, Factor 2 (F2) reflects an earlier constriction component likely driven by the pupillary light reflex, and Factor 3 (F3) represents an initial orienting response. Notably, the results in Figure 5 and the findings of the GAM analyses revealed that higher attractiveness ratings were associated with later dilation within the temporal window represented by F1. These findings suggest that more attractive stimuli elicit increased attention (Binda et al., 2013a; Bombeke et al., 2016; Mathôt et al., 2013; Naber et al., 2013) and increased arousal (Bradley et al., 2008; Kuraguchi & Kanari, 2020; Kuraguchi & Kanari, 2021; Laeng et al., 2013; Oliva & Anikin, 2018), consistent with previous findings. While pupil dilation has frequently been associated with high attractiveness, Liao et al. (2021) reported the opposite effect, namely, that transient pupil constriction occurs in response to highly attractive faces (Liao et al., 2021). Interestingly, a similar pattern of early constriction was observed in the present study within the early time window (500–1,500 ms) of F2, as revealed by the GAM analyses. This result is particularly notable given the correspondence between timing and response polarity. However, several methodological differences limit direct comparisons of these results. Specifically, facial stimuli were used in Liao et al. (2021), perceptual attributes and affective evaluations were not distinguished in the evaluative task, and raw pupil signals were analyzed without decomposing temporally distinct components. In contrast, we applied tPCA to images of general objects and directly compared perceptually grounded (glossiness) and affective (attractiveness) evaluations made by the same participants. Despite these differences, our findings suggest that automatic pupillary modulations may arise during early processing phases in response to visually attractive stimuli, even prior to the completion of later evaluative processing. Moreover, consistent with the results of Santos et al. (2023), our findings indicate that physiological responses can vary depending on the instructions provided in evaluative tasks, even when the same visual input is used across tasks (Santos et al., 2023). Importantly, while Santos et al. manipulated the evaluative context between participants, our within-subjects design allowed for a more direct comparison of pupil dynamics across tasks using identical stimuli.

In addition to the patterns observed for F1, the time-resolved analyses of F2 revealed differences in how pupil responses varied across ratings and tasks. Specifically, in the present study, higher glossiness ratings were associated with greater pupil constriction (Figures 3 and 5), which is consistent with the findings of Tamura et al. (2024). At the peak of light-induced pupillary constriction, which occurred approximately 1,000 ms after stimulus onset, the difference in pupil diameter between the lowest (rating 1) and highest (rating 7) glossiness levels reached approximately 0.15 mm. This value is comparable to or even exceeds the 0.1 mm effect size reported in a prior study (Tamura et al., 2024) (see the right panel in Figure 3). The magnitude of this modulation is also consistent with previous studies reporting pupil constriction in response to illusory brightness or high-level image content that alters subjective luminance perception (Binda, Pereverzeva, & Murray, 2013b; Laeng & Endestad, 2012; Naber & Nakayama, 2013). Notably, additional analyses of trial-averaged factor scores revealed no significant task × rating interaction, suggesting that this modulation is not specific to a particular task but rather reflects a shared component associated with the pupillary light reflex. Moreover, additional analyses based on a four-group categorization of stimuli (low/high glossiness × low/high attractiveness) revealed no reliable interaction between glossiness and attractiveness for any factor. A main effect of glossiness was observed for Factor 2, whereas no corresponding effect of attractiveness was found, suggesting that pupil responses in this time window are more closely related to glossiness-related properties than to attractiveness-related properties at the level of overall amplitude.

Furthermore, task-related differences in pupil responses were observed for F3. Given that the observed modulation occurred in a time window preceding the peak of the pupillary light reflex, it likely reflects a transient response associated with novelty or orienting mechanisms. As shown in Figure 5, pupil changes appear to be linked to attractiveness ratings. These results suggest that the attractiveness judgment task, which may be associated with higher emotional and social salience, could have induced the differential allocation of attentional resources through mechanisms potentially mediated by subcortical structures such as the superior colliculus (Wang, Boehnke, Itti, & Munoz, 2014; Wang & Munoz, 2015). However, because the proportion of the variance explained by this component was relatively low (9.6%), this effect should be interpreted with caution.

### Flexibility of pupil dynamics across task contexts

The tPCA results revealed systematic differences in the expression of latent components across tasks (glossiness vs. attractiveness), suggesting that pupil dynamics are not solely constrained by a fixed physiological manifold (Blini et al., 2024) but can be modulated depending on the cognitive context imposed by the task. These findings differ from the results described by Blini et al. (2024), who reported that pupillary responses to numerical stimuli followed a shared low-dimensional trajectory across different evaluative tasks, indicating a robust physiological structure that was largely invariant to cognitive framing. However, we note a key methodological distinction between the two studies in terms of how “stimulus constancy” was operationalized. In Blini et al.’s study, stimulus properties such as the numerical value and brightness varied across tasks, whereas we presented physically identical stimuli under different evaluative instructions. This approach allowed for a more controlled comparison of how the task context alone modulates pupil dynamics. Taken together, our results suggest that pupillary responses are more flexibly shaped by task demands than previously assumed and provide a complementary perspective on the idea of a fixed pupillary manifold. In particular, the results suggest that the temporal structure of the pupil response reflects not only stimulus-driven constraints but also differences in evaluative processing.

### Methodological considerations and control analyses

We controlled the luminance levels of all the stimuli to characterize the psychosensory components of the pupillary response. These components reflect processes beyond the luminance-driven light reflex, and our goal was to compare them across different evaluative tasks. However, recent studies have shown that pupillary responses can exhibit orientation-dependent modulations even when luminance is held constant (Parrella, Gurung, & Grubb, 2025). These findings suggest that despite maintaining uniform luminance levels across stimuli, subtle variations in the spatial configuration of the stimulus (such as whether the object is vertically or horizontally elongated) may still influence pupil size. Therefore, caution is warranted when interpreting psychosensory effects because spatial anisotropy in the stimulus layout could contribute to task-dependent pupil dynamics.

The observed differences in pupil responses between the glossiness and attractiveness tasks could also be explained by the fact that each evaluation was conducted in a separate block. Performing a series of judgments within the same task block may induce a task-specific evaluative mode or mental set, which could influence pupillary responses. Thus, we reanalyzed the data by including block order (attractiveness first vs. glossiness first) as a factor in LMMs computed based on temporally averaged factor scores to examine this possibility (Supplementary Figure S1). No main effect of the block order was observed for any factor. However, a significant task × rating × block order interaction effect was observed for all components. An inspection of this interaction revealed that the relationship between the ratings and pupil responses differed across tasks depending on the block order, with variations in both the baseline value and slope. This pattern is consistent with the interpretation that the observed differences are not solely attributable to stable task-specific effects but are modulated by the block-level task context. The effect was strongest for the later component (Factor 1) and weaker for the earlier components, which is consistent with the idea that later pupillary dynamics are more susceptible to higher-order cognitive influences such as the task set and contextual adaptation (e.g., Mathôt, 2018). Thus, while these results indicate that the primary findings cannot be reduced to a simple block-order confound, they suggest that the task context contributes to the observed modulation of pupil responses. Future studies with interleaved task designs would be valuable to further distinguish task demands from broader contextual influences.

In addition, we included the trial type (first vs. second repetition within a block) as a factor in analogous models to assess potential set formation effects within each block. A significant main effect of the trial was observed only for Factor 2, indicating a modest difference between repetitions. Crucially, this effect did not interact with the task or rating, suggesting that it reflects a general repetition-related adjustment (e.g., habituation) and does not indicate task-specific set formation. No reliable trial-related effects were observed for Factor 1 or 3. Importantly, the absence of interactions involving the trial indicates that the overall task × rating patterns were stable across repetitions, supporting the reliability of the observed pupillary responses.

We further examined whether the observed task-related differences could be attributed to differences in stimulus composition across rating levels by conducting additional analyses using explicitly matched stimulus subsets. Specifically, we repeated the LMM analyses on subsets in which the stimulus identity was constrained by restricting images to intermediate ranges of glossiness and attractiveness ratings. For the most stringent subset, we selected 20 images whose mean glossiness and attractiveness ratings both fell within the intermediate range (3–5), and this image set was analyzed under the glossiness and attractiveness task conditions. Across these subset analyses, no reliable main effect of the task was observed for any temporal component. In contrast, a main effect of the rating within each subset was observed for Factor 2. These findings indicate that the previously reported task-related differences cannot be reduced to differences in the specific images contributing to each rating level. Rather, they suggest that the observed effects are not driven by the stimulus identity and are better characterized in terms of temporally specific modulations of pupil responses.

We cannot rule out the possibility that the effects of glossiness and attractiveness overlap in time. However, even when such overlap is present, the components extracted from the shared covariance structure were associated with systematic differences in temporal weighting between tasks.

### Theoretical implications, limitations, and future directions

The present findings support the differential temporal weighting of glossiness and attractiveness judgments in pupil dynamics. Although we interpret this difference within a hierarchical processing framework, we emphasize that the underlying processing mechanisms cannot be localized directly on the basis of the present data. In principle, similar temporal differences could arise even between statistically independent features, provided that they differ in terms of the type or level of processing they engage. Thus, the hierarchical framework should be regarded as a theoretical interpretation rather than definitive evidence of distinct neural processing levels.

While the observed differences in the latency of the pupil responses, particularly the finding that attractiveness-related modulation emerged later than glossiness-related modulation did, can be interpreted by considering the hypothesis that surface property evaluations are hierarchically organized (Komatsu & Goda, 2018), we do not claim to directly test the underlying neural framework. Instead, our findings provide indirect physiological evidence that different types of material evaluations may involve temporally distinct processes. Moreover, we acknowledge that the present design does not allow us to isolate a strictly task-independent, purely stimulus-driven early time window. Although the hypothesis that an initial phase of the pupillary response primarily reflects automatic physiological reactions to the physical visual input is plausible, we could not explicitly define or test such a window with our data-driven approach.

Nevertheless, pupil diameter constitutes a one-dimensional physiological index. Although this measure is informative, it provides limited insight into the spatiotemporal dynamics of the underlying neural processes. In future research, multimodal approaches such as electroencephalography should be incorporated to better characterize the temporal dynamics of visual and affective evaluations, particularly in relation to surface perception (Orima, Wakita, & Motoyoshi, 2025; Wiebel, Valsecchi, & Gegenfurtner, 2014). However, importantly, electroencephalogram studies often focus on neural activity within the first 500 ms after stimulus onset, which differs from the temporal range typically analyzed by pupillometry. Thus, direct comparisons between the two modalities should be performed cautiously. Moreover, we focused exclusively on glossiness and attractiveness as representative examples of perceptually grounded and affective material attributes. Whether similar pupillary modulation patterns extend to other perceptual dimensions (e.g., roughness and transparency) or material categories (e.g., fabric or metal) remains an open question. Investigating pupillary responses across a broader range of attributes is essential for testing the generalizability of the present findings and refining theoretical models of material perception.

## Conclusions

In this study, we examined how pupillary responses differed when participants evaluated the glossiness and attractiveness of the same set of stimuli. tPCA and GAMs revealed that higher glossiness ratings were associated with greater pupil constriction during an earlier time window related to the light reflex. In contrast, higher attractiveness ratings were linked to increased pupil dilation emerging at later time points. These results may reflect differences in the temporal dynamics of processing engaged during each evaluative task. Overall, our findings indicate that task-dependent modulation of pupil size can elucidate the temporal structure of perceptual evaluation, contributing to a deeper understanding of how material perception occurs over time.

## Supplementary Material

Supplement 1
