# Supplementary material for: Task-dependent pupillary responses to glossiness and attractiveness judgments
Source: J Vis. 2026 Sep 1;26(9):1. doi: 10.1167/jov.26.9.1 (PMC13549414; doi:10.1167/jov.26.9.1)
Supplement: Supplementary file 1 [file jovi-26-9-1_s001.pdf]

Supplemental Information

Task-dependent pupillary responses to glossiness and attractiveness judgments

Hideki Tamura*, Shigeki Nakauchi, Tetsuto Minami

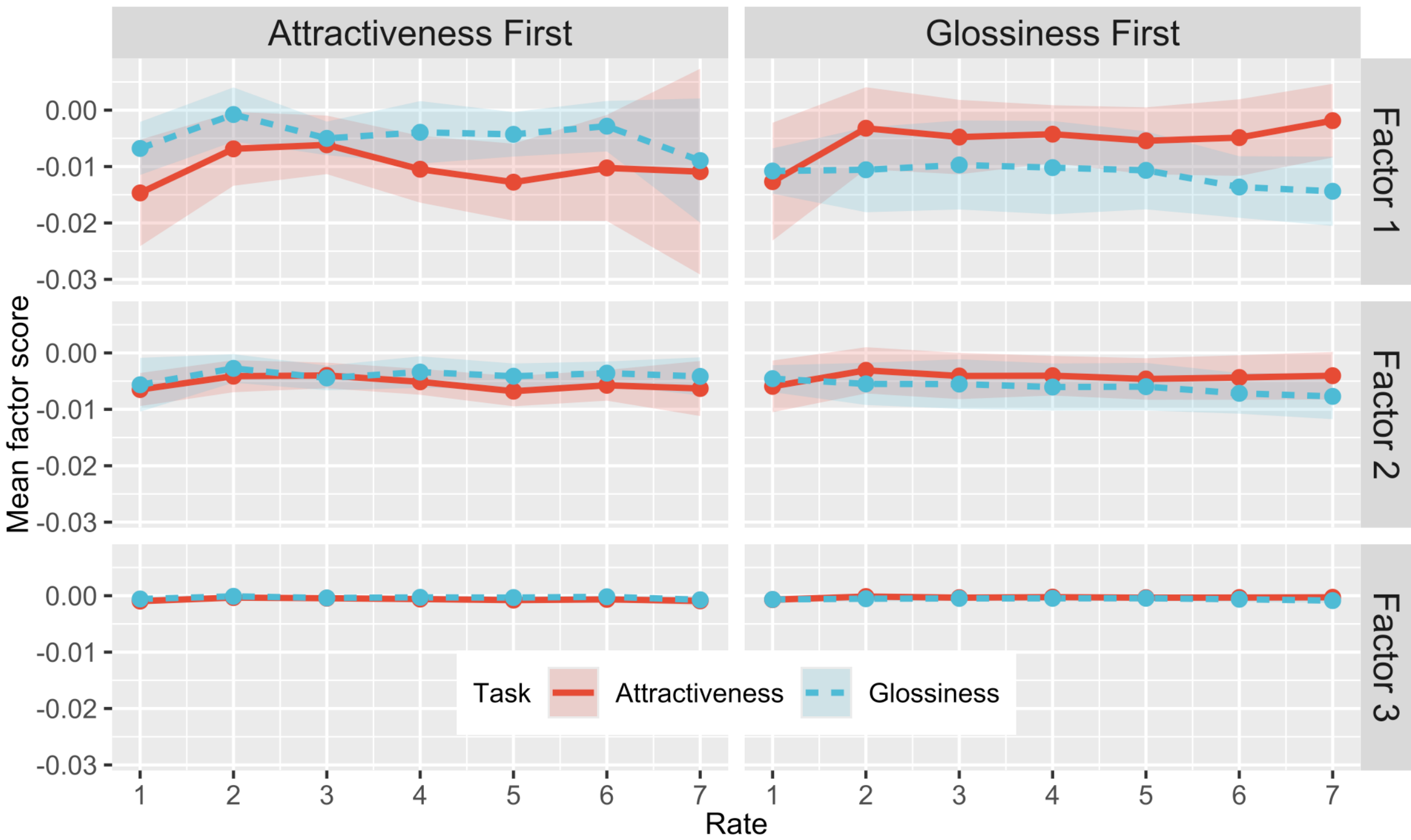

Figure S1. Effects of the task, rating, and block order on pupil component factor scores (F1–F3). The mean participant-level factor scores (±95% CI) are plotted as a function of the ratings separately for the attractiveness and glossiness tasks and for each block order (attractiveness first vs. glossiness first). Factor scores were computed by averaging time-resolved component scores across the stimulus presentation window (0–3000 ms) for each trial before model estimation was performed.

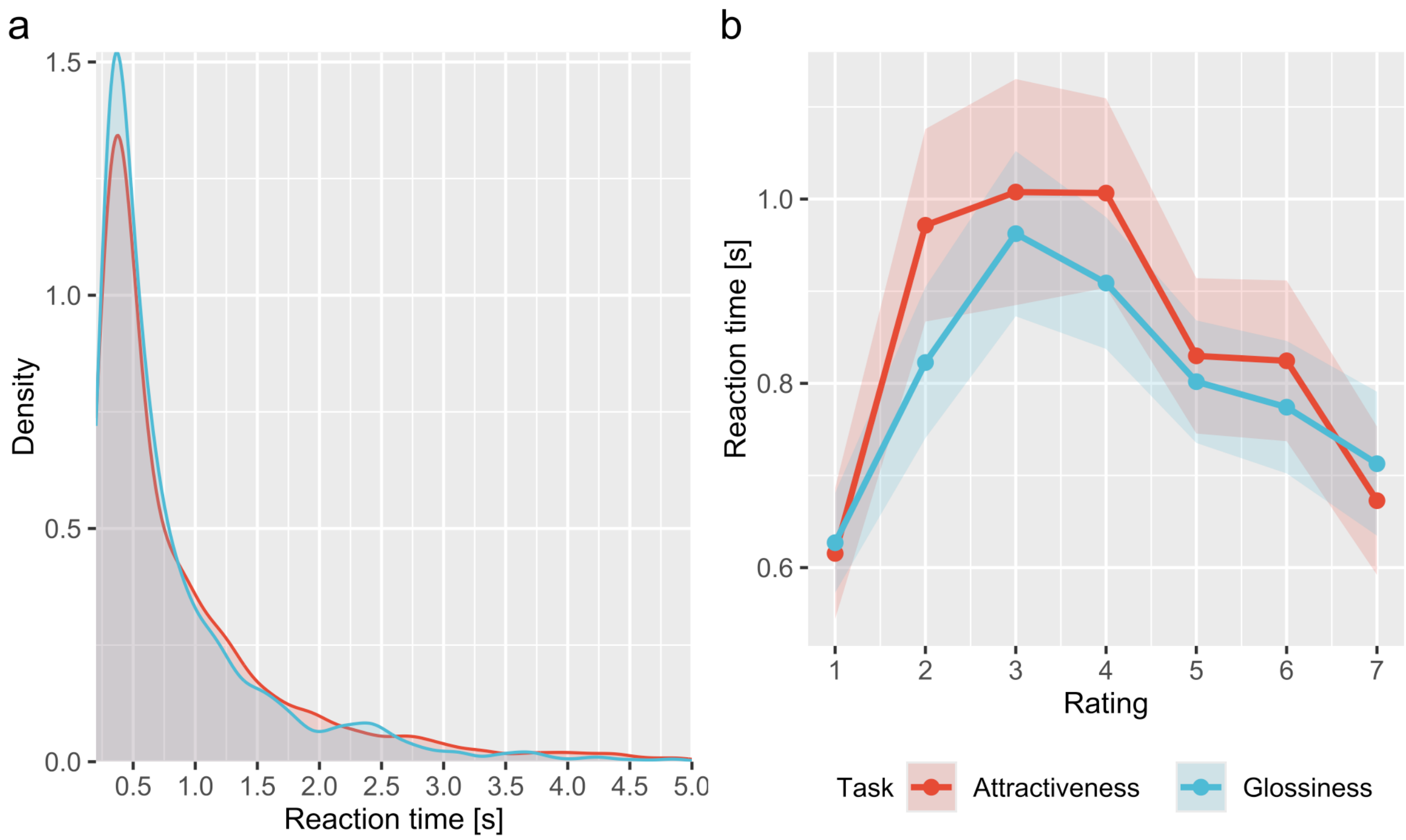

Figure S2. Reaction times. (a) Distribution of reaction times (RT) for each task. The density plots show the distribution of the trial-by-trial RTs separately for the attractiveness and glossiness tasks. In both tasks, a right-skewed distribution typical of RT data was observed, with broadly similar overall shapes. For visualization, trials with extremely fast (< 0.2 s) or slow (> 5 s) responses were excluded, which accounted for only a small proportion of the data (less than 2%). (b) The mean reaction time (RT) as a function of the rating level for each task. The points and shaded areas indicate the mean and 95% confidence intervals across participants, respectively. The RT exhibited an inverted-U relationship with the ratings, with slower responses for intermediate ratings than for extreme ratings. This pattern was more pronounced in the attractiveness task than in the glossiness task. All trials were included in this analysis.

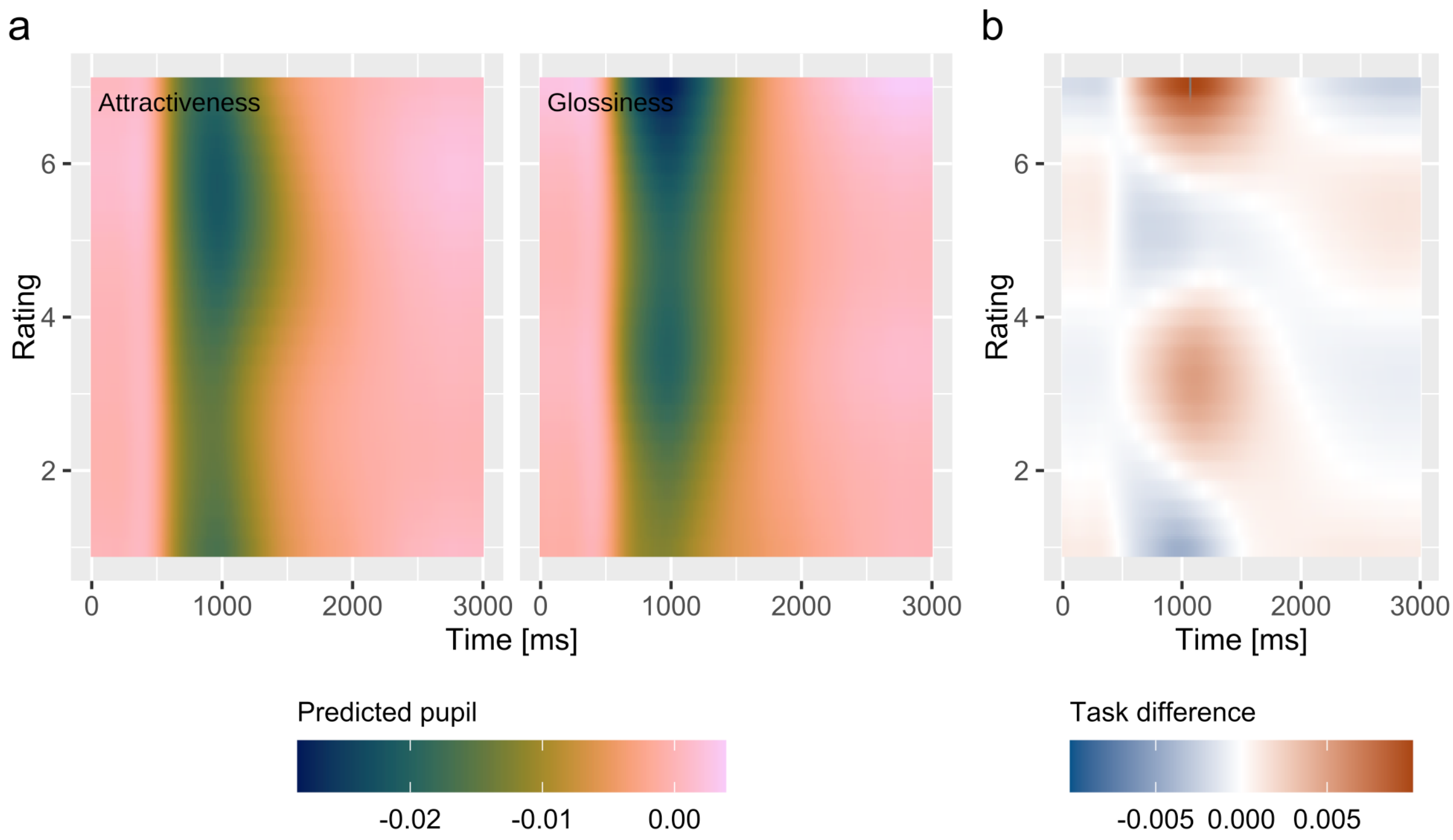

Figure S3. Predictions of the generalized additive model for Factor 2 across the continuous time × rating space. The format was the same as that of Figure 6 for Factor 1.

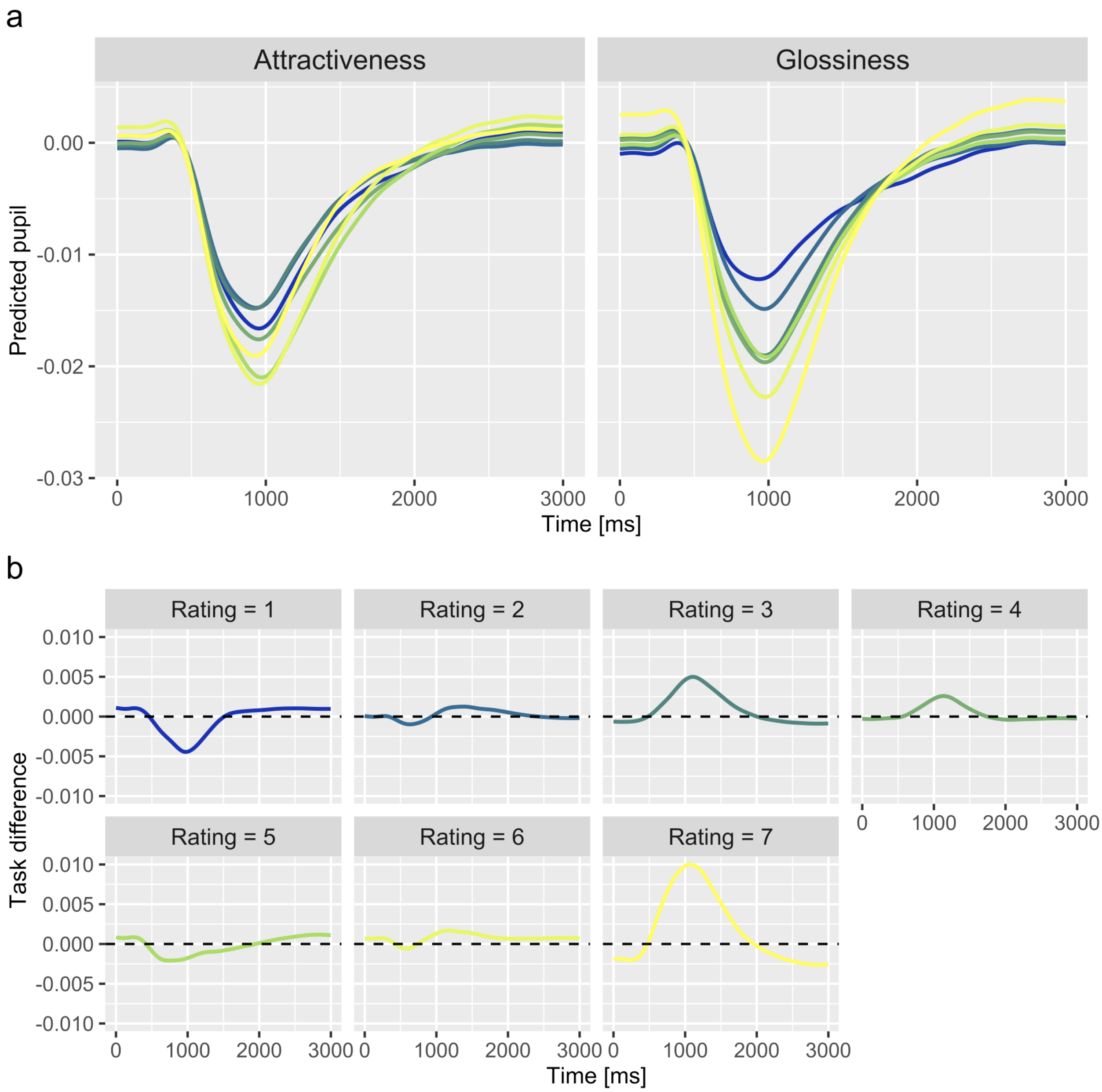

Figure S4. Time-resolved modulation of pupil responses as a function of the rating and task for Factor 2. The format was the same as that of Figure 7 for Factor 1.

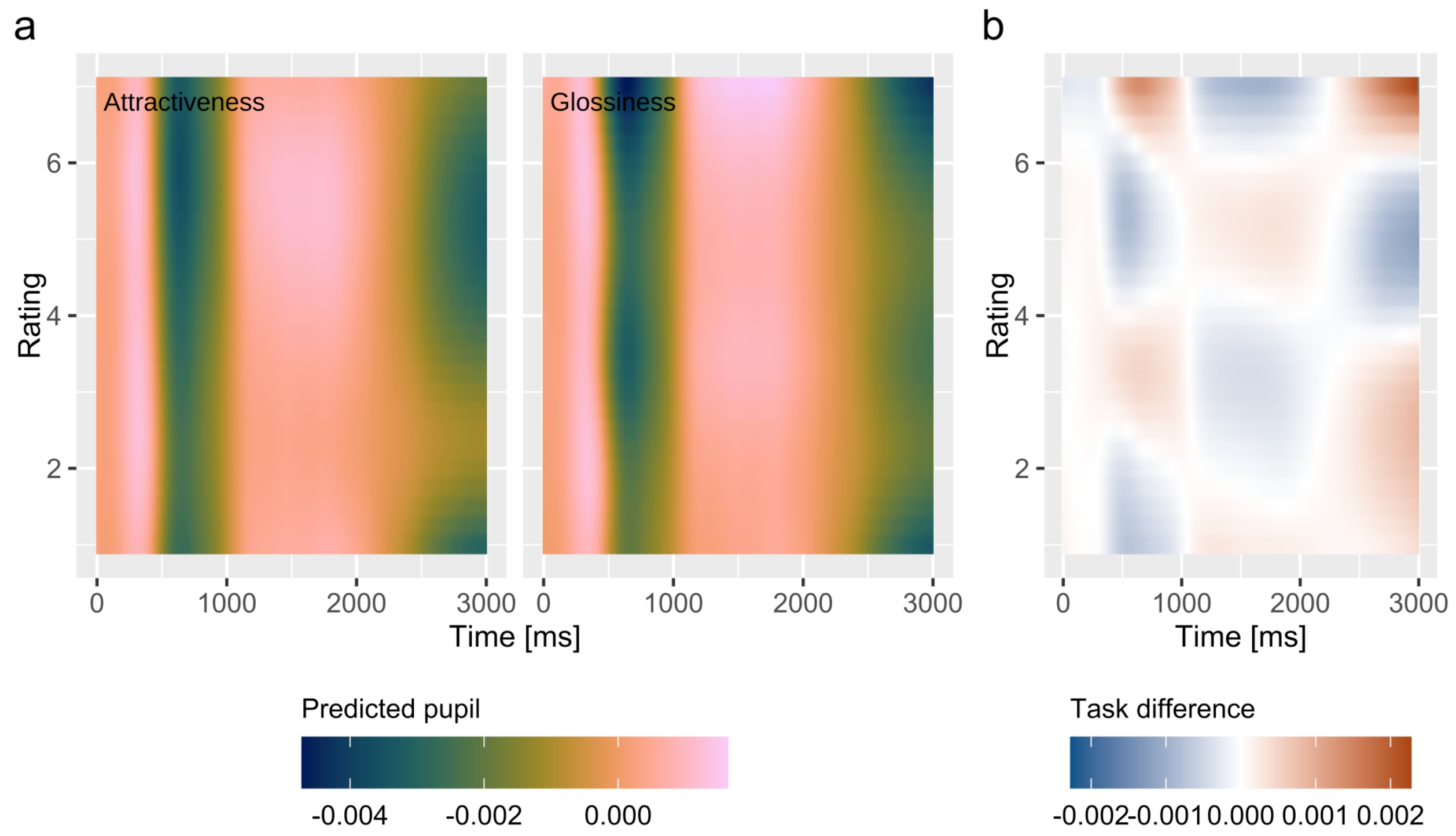

Figure S5. Predictions of the generalized additive model for Factor 3 across the continuous time × rating space. The format was the same as that of Figure 6 for Factor 1.

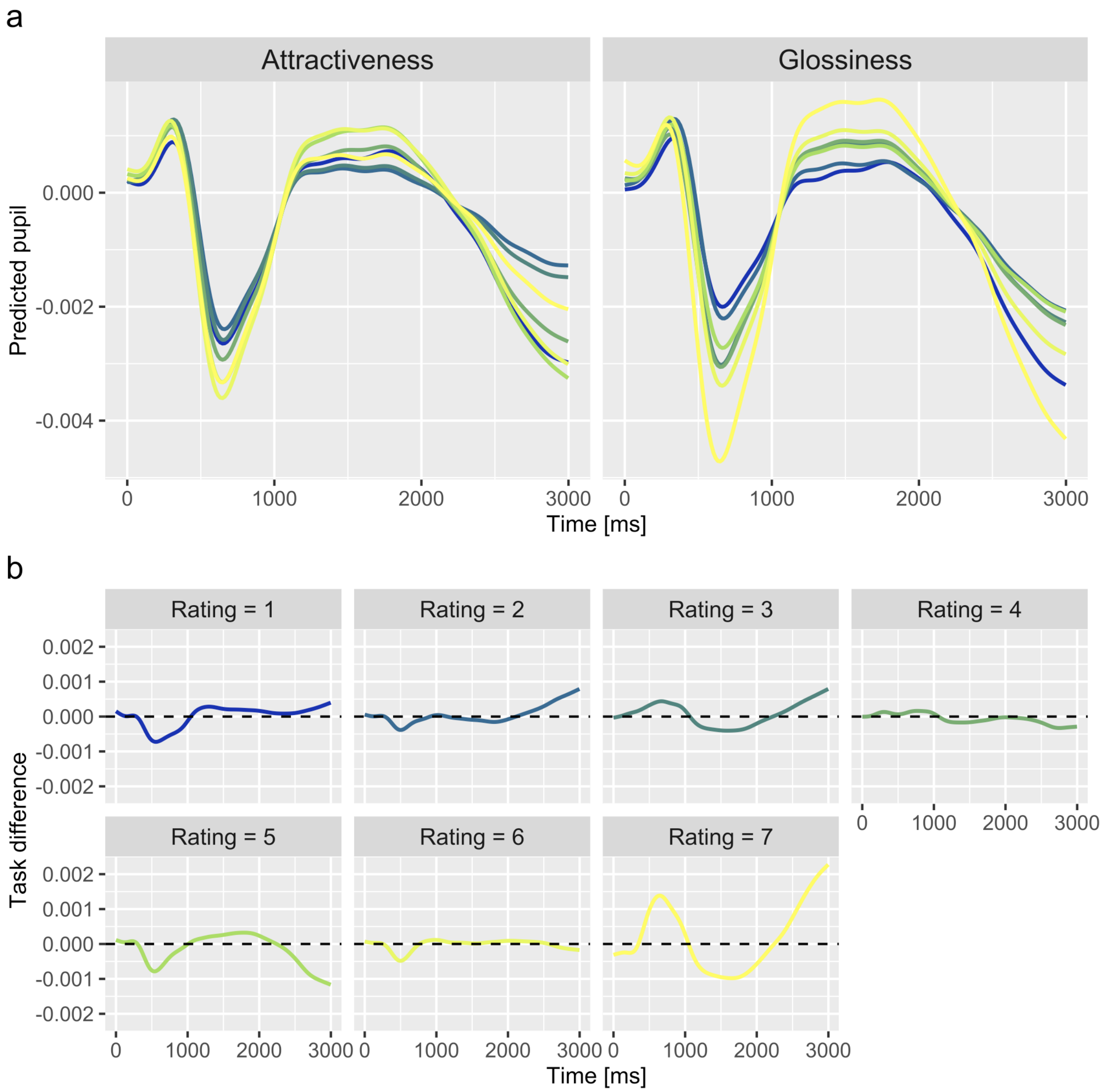

Figure S6. Time-resolved modulation of pupil responses as a function of the rating and task for Factor 3. The format was the same as that of Figure 7 for Factor 1.
